# Serum extracellular vesicles containing MIAT induces atrial fibrosis, inflammation and oxidative stress to promote atrial remodeling and atrial fibrillation via blockade of miR‐485‐5p‐mediated CXCL10 inhibition

**DOI:** 10.1002/ctm2.482

**Published:** 2021-08-03

**Authors:** Yingwei Chen, Xiaojie Chen, Haiyu Li, Yunpeng Li, Dong Cheng, Yi Tang, Haiqiang Sang

**Affiliations:** ^1^ Department of Cardiology The First Affiliated Hospital of Zhengzhou University Zhengzhou P.R. China

**Keywords:** atrial fibrillation, CXCL10, extracellular vesicles, fibrosis, inflammation, MIAT, microRNA‐485‐5p, oxidative stress

## Abstract

**Background:**

Atrial fibrillation (AF), a supraventricular arrhythmia that impairs cardiac function, is a main source of morbidity and mortality. Serum‐derived extracellular vesicles (EVs) have been identified to carry potential biomarker or target for the diagnosis and treatment of AF. We intended to dissect out the role of lncRNA MIAT enriched in serum‐derived EVs in AF.

**Methods:**

MIAT expression was quantified in EVs isolated from serum samples of AF patients. Mouse and cell models of AF were developed after angiotensin II (Ang II) induction. Relationship between MIAT, miR‐485‐5p, and CXCL10 was identified. Ectopic expression and depletion assays were implemented in Ang II‐treated mice or HL‐1 cells, or those co‐cultured with serum‐derived EVs to explore the roles of EV‐carried MIAT.

**Results:**

MIAT was upregulated in EVs from serum samples of AF patients. Further analysis indicated that MIAT enriched in serum‐derived EVs promoted atrial fibrosis, inflammation and oxidative stress, and aggravated the atrial remodeling and resultant AF. Mechanistically, MIAT bound to miR‐485‐5p and weakened its inhibitory role on the target CXCL10, which was responsible for the role of serum‐derived EV containing MIAT in cellular fibrosis, oxidative stress and inflammation, and atrial remodeling in vivo.

**Conclusions:**

In conclusion, serum‐derived EV containing MIAT facilitates atrial remodeling and exacerbates the AF by abolishing the miR‐485‐5p‐mediated CXCL10 inhibition. This finding aids in the deeper understanding about the pathophysiology of AF.

## Graphical abstract

EVs isolated from serum samples of AF patients carried MIAT to bind to miR‐485‐5p and caused the upregulation of the miR‐485‐5p target CXCL10, inducing atrial myocyte fibrosis, inflammation, and oxidative stress, thus promoting atrial remodeling and aggravating the AF.

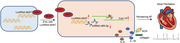

**Abbreviations**
AF: atrial fibrillation
Ang II: angiotensin II
CXCL10: C–X–C motif ligand 10
DCFH‐DA: dichloro‐dihydro‐fluorescein diacetate
DHE: dihydroethidine
EVs: extracellular vesicles
HE: hematoxylin‐eosin
si‐CXCL10: small interfering RNA‐targeting CXCL10

## 1 BACKGROUND

As a principle clinical cardiac arrhythmia, atrial fibrillation (AF) occurs upon suppression of the normal sinus mechanism by a diffuse and chaotic pattern of electrical activity in the atria.^1^ AF probably initiates tremendous complications with regard to the morbidity and mortality, particularly as it induces hemorrhage or stroke, imposing high global disease burden.^2^ Current strategies to tackle AF mainly focus on ablation therapy, anticoagulation, antiarrhythmic drugs, left atrial appendage obliteration and surgical treatments^3^; however, the incidence of AF recurrence is increasing.^4^ Identifying modulators of AF initiation and progression is thus highlighted to better develop prevention and treatment strategies.

Discovery of serum‐derived extracellular vesicles (EVs) opens up a new perspective in the complex mechanism underlying AF and provides potential biomarkers for assessing severity or prognosis of AF.^5^ EVs, known as membrane vesicles composed of exosomes and microvesicles, share interaction with the recipient cells and consequently deliver their bioactive contents, including proteins, lipids, and RNAs.^6^ Such a long noncoding RNA, MIAT is firstly extracted as a promising gene for myocardial infarction and later has also been identified as a candidate biomarker and therapeutic target in many malignant tumors due to its regulation in cell antiapoptosis, invasion, metastasis, and proliferation via various mechanisms.^7^ Moreover, MIAT upregulation has been observed in the EVs derived from serum samples of gastric cancer patients.^8^ In addition, MIAT induced by high glucose has been reported to present a dramatic increase in peripheral blood leukocytes of AF patients and responsible for cardiac fibrosis, cardiac contractility, and inflammation in cardiomyocytes.^9^ Strikingly, MIAT downregulation prominently alleviates AF as it can increase atrial effective refractory period while reducing duration of AF alongside cardiomyocyte apoptosis.^10^ lncRNAs function as a sponge for miRNAs and inversely orchestrate their expression through sequence‐specific binding.^11^ The miRWalk database predicted binding site between MIAT and microRNA (miR)‐485‐5p. miRNAs can serve as biomarkers to reflect the progression of AF owing to their involvement in atrial function, oxidative stress, and fibrosis pathways.^12^ miR‐485‐5p functions as an inhibitor of mitochondrial fission and hypertrophy triggered by phenylephrine, hence protecting against cardiac hypertrophy.^13^ miR‐485‐5p binding sites were also predicted in 3′UTR of C–X–C motif ligand 10 (CXCL10) by the RNA22 database. CXCL10 is known as a proinflammatory chemokine^14^ and its elevated levels have been observed in the AF patients.^15^ Thus, we speculated whether serum‐derived EVs could carry MIAT, which might play a critical part in AF development via mediating the miR‐485‐5p/CXCL10 axis. Therefore, in this study, we isolated EVs from serum samples from AF patients and mouse AF models and conducted co‐culture experiments to verify this hypothesis so as to provide effective therapeutic strategies combating AF.

## 2 MATERIALS AND METHODS

### 2.1 Ethics statement

The Ethics Committee of The First Affiliated Hospital of Zhengzhou University provided ratification for this research that was implemented strictly as per the *Declaration of Helsinki*. Before enrollment, all participants provided informed consents. Animal assays were conducted as per the Guide for the Care and Use of Laboratory animals published by the US National Institutes of Health.

### 2.2 Sample collection

Peripheral blood was harvested from 20 patients with AF who were treated in the Department of Cardiology of The First Affiliated Hospital of Zhengzhou University between June 2019 and November 2019, and from 20 healthy individuals with normal blood pressure as controls (Table S1). Before blood sampling, medical history of the healthy individuals (age‐ and gender‐matched individuals who have normal sinus rhythm, no family history of AF, and no significant physical abnormalities) and AF patients was carefully examined. Patients meeting the following criteria were enrolled in this research based on 2014 American Heart Association (AHA) guidelines: (a) patients were diagnosed with paroxysmal AF, that is, self‐terminating AF within 7 days persistent AF in which interventions are required for termination, or permanent AF in which sinus rhythm cannot be restored or maintained^16^ (there were six patients with paroxysmal AF, seven patients with persistent AF, and seven patients with permanent AF); (b) patients aged above 18 years; and (c) patients had normal ejection fraction (EF >55%). Besides, none of the patients had cardiomyopathy, congestive heart failure, thyroid disease, valvular heart disease, hematological system diseases, ischemic heart disease, and any other infectious or immune diseases (sepsis, antineutrophil cytoplasmic autoantibody‐associated vasculitis, plasmodium interstitial malaria, arthritis, inflammatory bowel disease, peptic ulcer and allergy induced by *Helicobacter pylori*).

### 2.3 Isolation and identification of EVs from serum samples of AF patients

Subsequent to transferring into BD Vacutainer SST II Advance Tubes (Becton, Dickinson and Company, Franklin lakes, NJ, USA), bloods underwent coagulation at room temperature for at least 1 h and 20 min centrifugation at 3000*g* and 10°C to discard cells. After being attained into a new Eppendorf tube, the supernatant received 30‐min centrifugation at 10,000*g* and 4°C to discard debris and large vesicles. Next, 1‐ml serum aliquots were diluted to 8 ml with DPBS without Ca2+ or Mg2+ and centrifuged at 100,000*g* and 4°C (polycarbonate tube, fixed angle T‐1270 rotor, Thermo Fisher Scientific Inc., Waltham, MA, USA). EV precipitate of all patients was collected, resuspended using 100‐μl DPBS and subsequently stored at −80°C for further experiments.

Thereafter, the isolated EVs were imaged under a transmission electron microscope (TEM). Briefly, EV suspension was mixed with equal volume of 4% paraformaldehyde, and 5 μl mixture was supplemented to carbon‐coated grids, and then dyed with 1% phosphotungstic acid. Images were obtained under a TEM (HT7830, Hitachi Chemical, Tokyo, Japan).

Nanoparticles tracking analysis was then utilized to analyze the size. EVs were resuspended in 1 ml PBS with filtered PBS as a control. The diluted EVs were then injected into the NanoSight LM10 (NanoSight Ltd., Minton Park, UK) to measure the size at the temperature of 23.75°C ± 0.5°C for 60 s.

**HIGHLIGHTS**

1. Serum‐derived EVs containing MIAT promote atrial myocyte fibrosis, inflammation, and oxidative stress.
2. MIAT binds to miR‐485‐5p to upregulate CXCL10.
3. CXCL10 overexpression facilitates atrial myocyte fibrosis, inflammation, and oxidative stress.
4. Serum‐derived EVs accelerate atrial myocyte fibrosis, inflammation, and oxidative stress in vitro and Ang II‐caused AF and atrial remodeling in vivo via MIAT/miR‐485‐5p/CXCL10 axis.

Western blot analysis was conducted to assess EV surface marker proteins. EVs were dissolved using RIPA buffer, followed by quantification utilizing a BCA kit (A53226, Thermo Fisher Scientific). All antibodies (Abcam Inc., Cambridge, UK; 1:1000) utilized included CD63 (ab134045), TSG101 (ab125011), and CD9 (ab92726).

### 2.4 Establishment of angiotensin II (Ang II)‐induced AF mouse models and animal treatment

Healthy male C57BL/6 WT mice (aged 6–8 weeks; Hubei Centers for Disease Control and Prevention) were housed in an animal experimental center with ad libitum access to food and water. Subsequent to 1‐week acclimation, mice were adopted for the experiment.

The mice were then treated with normal saline and Ang II (dissolved in 0.01% acetic acid; 2000 ng/kg/min; Sigma‐Aldrich). Next, mice were induced with a microosmotic pump (0.11 μl/h, Alzet model 1004, Alza Corp., Palo Alto, CA) for 3 weeks. The success of the model establishment was assessed by detecting systolic blood pressure (SBP) using a Softron BP98A tail cuff system (Softron Tokyo, Japan).

Mice underwent anesthesia using 2.5% tribromoethanol (a dose of 0.02 ml/g; acquired from Sigma‐Aldrich) for electrophysiology. The whole experiment was conducted at 37.0°C ± 0.5°C. With the temperature of mice controlled using a mouse circuit board (JRD‐7w, Nomoypet, Jiaxing, China) with heating elements, Millar 1.1 F octapolar EP catheter (Scisense, USA) was inserted into mice from right jugular vein and pushed into right atrium and ventricle for intracardiac pacing. Lead surface electrocardiograms and up to four intracardiac bipolar electrograms (GY6328B, Huanan Medical Science and Technology Co., Ltd., Henan, China) were documented by a computer‐based data acquisition system. The inducibility was assessed using an automatic stimulator, a part of the data acquisition system (GY6328B, Huanan Medical), and applying 5 s of burst to the catheter electrode. Burst pacing started with a cycle of 40 ms, then declined by 2 ms during each successive burst until the cycle intensity was 20 ms, and performed three times in each mouse. If at least two of the three pacing tests produced AF, the inducibility was considered to be positive. A rapid irregular rhythm lasting at least 1 s was suggestive of successful AF induction.

A total of 100 μl of mouse serum‐derived EVs containing DPBS was injected into Ang II‐induced mice via tail vein once a week with the final concentration of 3 μg/μl for 3 weeks.^17^, ^18^ In animal experiments, lentivirus transfection was adopted for gene editing, and CXCL10 was silenced by pGMLV plasmid. Primer sequence, vector construction, virus packaging and purification were completed by Sangon Biotechnology, and the experimental steps were carried out as per the instructions. Animals were injected at the fixed point. Before Ang II injection, mice were administrated with 100 μl 5 × 10^9^ plaque‐forming units (PFU) adenovirus‐packaged pGMLV‐MIAT (MIAT overexpression plasmid), small interfering RNA‐targeting CXCL10 (si‐CXCL10) or si‐NC (Sangon) via tail vein. All adenoviruses were purchased from Sangon Biotechnology.

### 2.5 SBP measurement

SBP was measured in restrained conscious mice. An automatic system with photoelectric sensors and a dual channel recorder (BP‐98A, Softron), with tail cuff and sphygmomanometer connected, were applied for indirect blood pressure measurements that were closely related to direct arterial measurements. Each mouse was measured thrice, with the average value recorded.

### 2.6 Collection of mouse blood samples

After the last induction of AF, blood samples of mice were collected in the ophthalmic venous plexus with capillaries and allowed for coagulation at room temperature for at least 1 h. EVs were then isolated from the blood samples of mice as above.

### 2.7 Masson's trichrome staining

Subsequent to anesthesia of mice through intraperitoneal injection of overdose pentobarbital (100 mg/kg), atrial tissues were quickly immobilized, paraffin‐embedded, and cut into sections (5 μm). The total collagen content in lung tissues was measured by staining with Masson's trichrome. The deposition of collagen in lung tissue in airway wall was finally observed under a microscope, where the collagen fiber was dyed in blue, cytoplasm of muscle fibers in red, and nucleus in blue brown.

### 2.8 Hematoxylin‐eosin (HE) staining

Atrial tissue sections were put onto the slide, dewaxed, and rehydrated in descending series of alcohol. Following 5‐min washing with distilled water, the sections were dyed with hematoxylin. The sections received differentiation in 1% hydrochloric acid ethanol and eosin staining before dehydration, clearing, and sealing. Observation was implemented under an optical microscope in 10 random visual fields from each slice, with the images obtained by the image acquisition system.

### 2.9 Immunohistochemistry

Paraffin‐embedded atrial tissues were sliced (4 μm). Following antigen retrieval, slice was added with 0.3% H_2_O_2_ for 10 min. Following blocking with 5% BSA, slices were immunostained with primary Mac‐2 antibody (1:200, sc‐374541, Santa Cruz Biotechnology) at 4°C overnight. Afterwards, slices underwent 20‐min re‐immunostaining with biotinylated secondary goat anti‐mouse IgG antibody (1:2500, ab6789, Abcam) and 20‐min cultivation with HRP‐streptavidin reagents (Innova Biosciences, Cambridge, UK). Thereafter, slices were developed with DAB before hematoxylin counterstaining. Slices were imaged utilizing a Leica‐DM2500 microscope (Leica‐DM2500, Germany) and quantified by the ImagePro Plus 7.1 software (Media Cybernetics).

### 2.10 Dihydroethidine (DHE) staining

The production of superoxide in atrial myocytes was detected by in situ DHE kit (Invitrogen Molecular Probes, Eugene, OR, USA). Paraffin‐embedded mouse atrial tissues were made into frozen slices (5 μm) at −80°C. Slices were then dyed with DHE reaction mixture and examined using a laser confocal microscope (Olympus FV10C‐W3). Finally, the fluorescence intensity was examined using the ImageJ software.

### 2.11 Dichloro‐dihydro‐fluorescein diacetate (DCFH‐DA) assay

HL‐1 cells (Procell Life Science&Technology Co., Ltd., Wuhan, China) were added with diluted DCFH‐DA mixture (1 ml) before 20‐min incubation at 37°C. Then cells were rinsed with medium without serum thrice so as to remove the DCFH‐DA failing to enter the cells. Olympus FV10C‐W3 laser confocal microscope was used to observe cells and photograph. Following cell collection, a microplate reader (SpectraMax M5) was applied for fluorescence intensity measurement (485 nm excitation wavelength and 535 nm emission wavelength).

### 2.12 Cell incubation and treatment

Mouse atrial muscle cell line HL‐1 received incubation in Claycomb medium (Sigma‐Aldrich) encompassing 100 U/ml streptomycin, 2 nM L‐glutamine, 10% FBS (Hyclone Laboratories, Logan, UT, USA), and 0.1 mM norepinephrine in a 37°C incubator with 5% CO_2_. Cells were seeded into a culture dish precoated with 0.02% gelatin and 12.5 μg/ml fibronectin, with medium daily renewed.

Next, the cells were treated with 100 ng of Ang II,^19^ and further with EVs (50 μg/ml),^18^ pGMLV‐MIAT, si‐MIAT, pGMLV‐CXCL10 (CXCL10 overexpression plasmids), miR‐485‐5p mimic, pGMLV‐MIAT + miR‐485‐5p mimic, EVs + si‐CXCL10, pGMLV‐MIAT + si‐CXCL10, pGMLV‐Vector, si‐NC, mimic NC, pGMLV‐MIAT + mimic NC, EVs + si‐NC, or pGMLV‐MIAT + si‐NC, respectively. At 48 h posttransfection, the following assays were implemented. The plasmid concentration was 50 ng/ml. The siRNA plasmid was constructed by Sangon Biotechnology.

### 2.13 RT‐qPCR

Total RNA extraction was implemented in tissues with TRIzol reagents (16096020, Thermo Fisher Scientific Inc.). For the detection of mRNA and lncRNA, cDNA was generated from the extracted RNA utilizing reverse transcription kits (RR047A, Takara). cDNA synthesis was achieved by PolyA Tailing test kit (B532451, Sangon) for miRNA detection. RT‐qPCR was conducted by the SYBR Premix Ex Taq II kit (DRR081, Takara) on an ABI 7500 instrument (Applied Biosystems). With U6 worked as a normalizer for miRNA, cel‐miR‐39 for the miRNA in EVs and GAPDH for the remaining genes, the fold changes were calculated utilizing 2^−ΔΔCt^ method. All primers are manifested in Table S2.

### 2.14 ELISA

Atrial tissues were homogenized and centrifuged, with the supernatant collected. IL‐1β and IL‐6 levels were detected utilizing an ELISA kit (Dakewe Biotech Company), followed by determination of OD values at 450 nm using ELISA instrument.

### 2.15 Western blot analysis

Total protein isolation was conducted in cells or tissues utilizing RIPA buffer (C0481, Sigma‐Aldrich), with the concentration estimated using the BCA kit (23227). Protein was uploaded onto SDS‐PAGE and electroblotted to PVDF membranes that received 1‐h blocking using BSA (5%) at ambient temperature. Membranes received overnight probing with primary antibodies at 4°C before 1.5‐h re‐probinh with HRP‐tagged goat anti‐rabbit IgG (1:20000, ab205718, Abcam) or goat anti‐mouse IgG (1:20,000, ab197767, Abcam) at room temperature. Next, developing solution (NCI4106, Pierce Biotechnology Inc., Rockford, IL, USA) was added to membranes, and band intensities were quantified using ImageLab software (Bio‐Rad Laboratories). The ratio of the gray value of the target band to that of internal reference protein GAPDH was representative of the relative protein expression of target genes. In this assay, the used primary antibodies consisted of antibody from Santa Cruz Biotechnology, Inc. to CXCL10 (mouse, 1:500, sc‐374092), and antibodies from Abcam to collagen I (rabbit, 1:1000, ab34710), collagen III (rabbit, 1:2000, ab7778), NOX2 (rabbit, 1:5000, ab129068), NOX4 (rabbit, 1:1000, ab154244), AGO2 (rabbit, 1:1000, ab156870), apolipoprotein B‐100 (apoB‐100) (1:1000, ab231574), and GAPDH (rabbit, 1:5000, ab194486).

### 2.16 Uptake of EVs

The separated EVs from serum were incubated with the red fluorescent dye PKH26 (acquired from Sigma‐Aldrich) for 5 min at ambient temperature. Subsequent to 90‐min centrifugation at 100,000*g*, EVs were suspended in basic medium, incubated with HL‐1 cells at 37°C for 12 h, and then immobilized with 4% paraformaldehyde. Treatment with equal amounts of EV‐free basic medium was employed as the control. The nuclei were dyed with 10 μg/ml Hoechst 33342 (C1025, Beyotime) for 10 min. The uptake of EVs by HL‐1 cells was observed through LSM710 laser scanning microscopes (LSM710, Carl Zeiss, Oberkochen, Germany) at excitation wavelengths of 350 and 551 nm.

### 2.17 EdU assay

HL‐1 cells were incubated into 96‐well plates (5 × 10^3^ cells/well) with 10 μM EdU solution for 24 h. Subsequent to immobilizing, cells were dyed with the use of Hoechst 33342 and analyzed under a fluorescence microscope (Thermo Fisher Scientific Inc.) at 350 and 550 nm. HL‐1 cell proliferation was evaluated by counting EdU‐positive cells.

### 2.18 Scratch test

HL‐1 cells were first incubated into a six‐well plate (5 × 10^5^ cells/well) overnight with 10% FBS‐contained medium until reaching 80%–90% confluence. Then the scrape wound was created using the pipette tip, and medium was renewed with one without serum. Images were recorded under a microscope and the scratch distance (0 h) was calculated.

### 2.19 Dual‐luciferase reporter assay

Then WT and MUT sequences of MIAT‐3′UTR and CXCL10‐3′UTR were constructed, followed by endonuclease cleavage of pmiR‐RB‐REPORTTM plasmid (acquired from RiboBio, Guangdong, China). Next, synthesized WT and MUT target gene fragments were inserted into the pmiR‐RB‐REPORTTM vector (RiboBio) with miR‐485‐5p, respectively. Dual‐Luciferase Reporter Assay System (E1910, Promega) was employed for measurement of luciferase activity. Sequence of CXCL10‐3′UTR‐WT was GGAUGGACAGCAGAGAGCCUCCU and that of CXCL10‐3′UTR‐MUT was GGAUGGACAGCAGAGUCGGAGA.

### 2.20 RIP assay

A RIP kit (Millipore) was employed. Cells underwent lysing with RIPA buffer (P0013B, Beyotime) before 10 min of centrifugation at 14,000 rpm and 4°C. Next, supernatant was harvested, a portion of which was removed as input, whereas the other was probed with rabbit anti‐human antibodies (Abcam) to Ago2 (ab186733, 1:50) and IgG (ab109489, 1:100, taken as NC) for coprecipitation. Finally, immunoprecipitated RNA was isolated and analyzed by RT‐qPCR.

### 2.21 RNA pull‐down assay

Cell transfection was implemented with 50 nM biotin‐labeled Bio‐miR‐485‐5p‐WT and Bio‐miR‐485‐5p‐MUT (GeneCreate, Wuhan, China). Cell lysates received overnight culture with M‐280 streptaviden magnetic beads (S3762, Sigma‐Aldrich) precoated with RNase‐free BSA and yeast tRNA (TRNABAK‐RO, Sigma‐Aldrich) at 4°C, before RT‐qPCR determination of MIAT enrichment. The sequence of Bio‐miR‐485‐5p‐WT was CUUAAGUAGUGCCGGUCGGAGA and that of Bio‐miR‐485‐5p‐MUT was CUUAAGUACCCAGCCAGCCUCC.

### 2.22 Statistical analysis

The measurement data described as mean ± standard deviation and SPSS 21.0 software (IBM Corp. Armonk, NY, USA) was adopted to analyze the data. The statistical significance was measured using unpaired *t*‐test (comparison between two groups), and one‐way ANOVA with Tukey's multiple comparisons test or repeated measures ANOVA with Bonferroni post hoc test (multiple comparisons); *p *< .05 was statistically significant.

## 3 RESULTS

### 3.1 Identification of serum‐derived EVs and high MIAT expression in serum‐derived EVs

**FIGURE 1:**
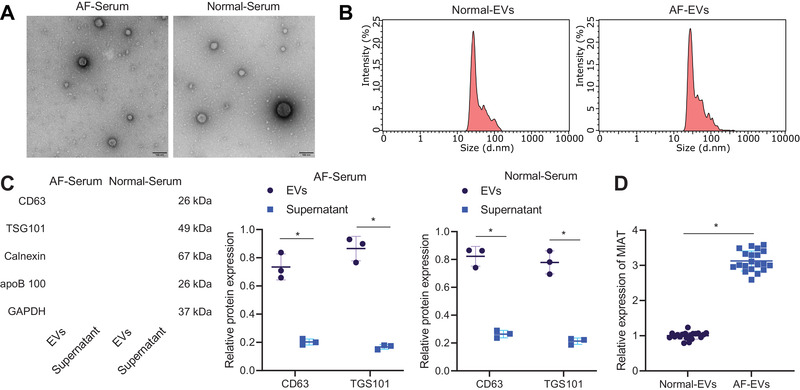
MIAT expresses highly in the serum‐derived EVs. (A) Morphological characterization of EVs observed using a TEM (scale bar = 100 nm). (B) Size distribution of EVs analyzed by nanoparticles tracking analysis. (C) Western blot analysis of EV surface maker proteins. (D) Expression of MIAT determined by RT‐qPCR in the EVs from serum samples of AF patients (*n* = 20) and normal individuals (*n* = 20). *p < .05. Data between two groups were analyzed by unpaired *t*‐test

We first identified the isolated EVs from patient serum samples. TEM observation showed that the serum‐derived EVs were round or oval membranous vesicle‐like in appearance (Figure 1A), and the size was 30–150 nm following nanoparticles tracking analysis (Figure 1B). Western blot analysis further demonstrated that the serum‐derived EVs were positive for CD63 and TSG101, but negative for Calnexin and apoB‐100 (Figure 1C). Therefore, the results suggested successful serum‐derived EV isolation. RT‐qPCR results further showed higher MIAT expression in EVs derived from serum samples of AF patients than those from serum samples of normal individuals (Figure 1D). Furthermore, the expression of MIAT was potently different in EVs from serum samples of patients with paroxysmal AF, persistent AF, and permanent AF, with the highest expression observed in the EVs from serum samples of permanent AF patients (Table S3). These results suggested that MIAT was present in serum EVs and was significantly highly expressed in serum EVs from patients with persistent AF.

### 3.2 Serum‐derived EVs containing MIAT promote atrial fibrosis, inflammation, and oxidative stress

**FIGURE 2:**
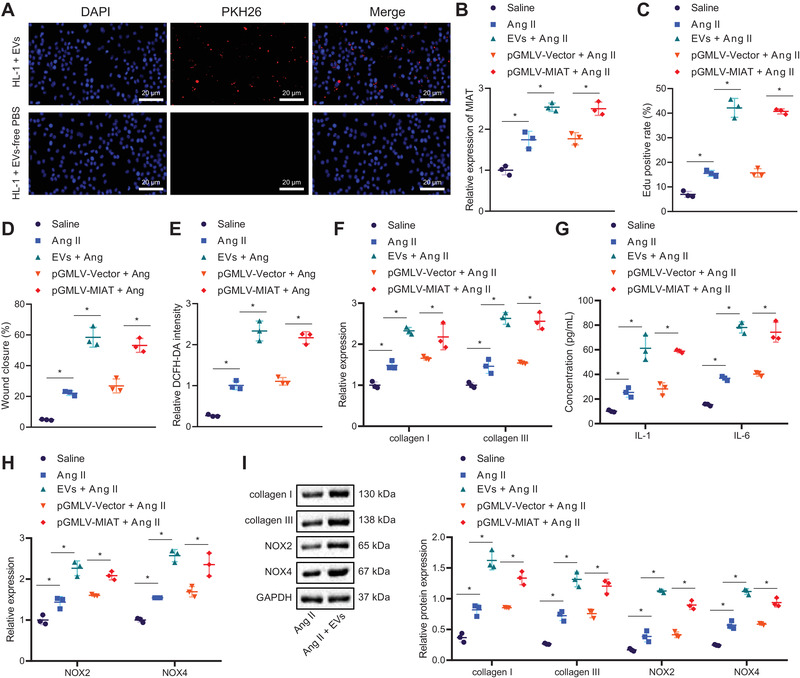
Serum‐derived EVs carrying MIAT trigger atrial fibrosis, inflammation, and oxidative stress. (A) Uptake of serum‐derived EVs by HL‐1 cells labeled by PKH26 (scale bar = 20 μm). HL‐1 cells were treated with PBS, or Ang II‐induced HL‐1 cells were treated or not treated with EVs, pGMLV‐Vector, or pGMLV‐MIAT. (B) Abundance of MIAT determined by RT‐qPCR in HL‐1 cells. (C) Proliferation of HL‐1 cells measured by EdU assay. (D) Migration of HL‐1 cells measured by Scratch test. (E) Production of superoxide in HL‐1 cells measured by DCFH‐DA. (F) Expression of collagen I and collagen III determined by RT‐qPCR in HL‐1 cells. (G) Expression of IL‐1β and IL‐6 in the supernatant of HL‐1 cells measured by ELISA. (H) Expression of NOX2 and NOX4 determined by RT‐qPCR in HL‐1 cells. (I) Western blot analysis of collagen I, collagen III, NOX2, and NOX4 proteins in HL‐1 cells. *p < .05. Data between two groups were analyzed by unpaired *t*‐test. Cell experiments were repeated three times independently

We then aimed to dissect out the effect of serum‐derived EVs containing MIAT on atrial myocytes. Figure 2A shows no fluorescence in the control cells treated with EV‐free basic medium and obvious red fluorescence in cells treated with EVs, suggesting that HL‐1 cells could uptake EVs. As reflected by RT‐qPCR, the abundance of MIAT was higher in HL‐1 cells treated with serum‐derived EVs than cells treated with PBS (Figure 2B). In addition, increased abundance of MIAT was also observed after Ang II‐induced HL‐1 cells received treatment with EVs or pGMLV‐MIAT. The effect of EVs was similar to that of pGMLV‐MIAT (Figure 2B). Meanwhile, the proliferation (Figure 2C) and migration (Figure 2D) of Ang II‐induced HL‐1 cells were accelerated and superoxide production (Figure 2E) was increased, which was further promoted following further treatment with EVs or pGMLV‐MIAT. Data of RT‐qPCR showed an increase of collagen I, collagen III, NOX2, and NOX4 expression in Ang II‐induced HL‐1 cells, which was further increased by EVs or pGMLV‐MIAT (Figure 2F,H). Moreover, IL‐1β and IL‐6 expression was upregulated in supernatant of Ang II‐induced HL‐1 cells, and treatment with EVs or pGMLV‐MIAT caused elevation of IL‐1β and IL‐6 expression in supernatant of Ang II‐induced HL‐1 cells (Figure 2G). Besides, Western blot analysis exhibited elevated collagen I, collagen III, NOX2, and NOX4 protein expression in Ang II‐induced HL‐1 cells, which was further enhanced by EVs or pGMLV‐MIAT (Figure 2I). The above results suggested that MIAT enriched in serum‐derived EVs could be internalized by atrial myocytes, and then promoted atrial fibrosis, inflammation, and oxidative stress.

### 3.3 Serum‐derived EVs containing MIAT facilitates Ang II‐caused AF and atrial remodeling

**FIGURE 3:**
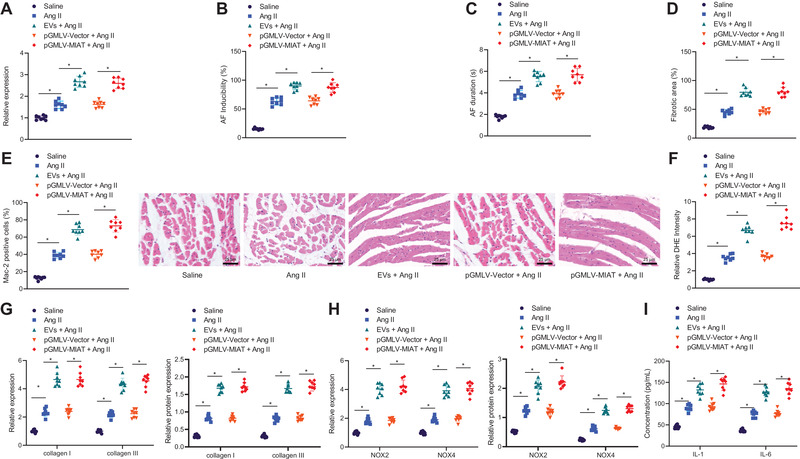
Serum‐derived EVs induce Ang II‐induced AF and atrial remodeling by delivering MIAT. Normal saline‐treated mice were utilized as control, and Ang II‐induced mice were treated with serum‐derived EVs, pGMLV‐Vector, or pGMLV‐MIAT. (A) Expression of MIAT determined by RT‐qPCR in atrial tissues of Ang II‐induced mice. (B) AF induction rate of Ang II‐induced mice. (C) AF duration of Ang II‐induced mice. (D) Masson's trichrome staining of fibrosis in atrial tissues of Ang II‐induced mice. (E) HE staining of pathological situations in atrial tissues of Ang II‐induced mice (scale bar = 25 μm) and immunohistochemistry for Mac‐2‐positive cells in atrial tissues of Ang II‐induced mice. (F) DHE staining of superoxide production in atrial tissues of Ang II‐induced mice. (G) Expression of collagen I and collagen III determined by RT‐qPCR and Western blot analysis in atrial tissues of Ang II‐induced mice. (H) Expression of NOX2 and NOX4 determined by RT‐qPCR and Western blot analysis in atrial tissues of Ang II‐induced mice. (I) Expression of IL‐1β and IL‐6 in atrial tissues of Ang II‐induced mice measured by ELISA. *n* = 8 for mice upon each treatment. *p < .05. Data among multiple groups were analyzed using one‐way ANOVA with Tukey's multiple comparisons test

Next, we shifted our attention to determine whether serum‐derived EVs aggravate AF and atrial remodeling by carrying MIAT in vivo. RT‐qPCR results showed an increase of MIAT expression in atrial tissues of Ang II‐induced mice treated with EVs or pGMLV‐MIAT (Figure 3A). The SBP of mice induced by Ang II was increased and additional EV treatment induced a more pronounced increase of SBP. In addition, compared to Ang II‐induced mice treated with pGMLV‐Vector or serum‐derived EVs, a higher SBP was observed in Ang II‐induced mice following MIAT overexpression (pGMLV‐MIAT) (Figure S1A). AF induction rate and AF duration were increased in Ang II‐induced mice, which showed a more pronounced increase in response to treatment with EVs or pGMLV‐MIAT (Figure 3B,C). In addition, treatment with EVs or pGMLV‐MIAT contributed to enhancement of Ang II‐induced atrial fibrosis (Figure 3D), inflammatory cell infiltration, Mac‐2‐positive macrophages (Figure 3E), and superoxide production (Figure 3F). Moreover, an elevation in the expression of collagen I, collagen III, IL‐1β, IL‐6, NOX2, and NOX4 was detected in atrial tissues of Ang II‐induced mice, while EVs or pGMLV‐MIAT led to a more significant elevation (Figure 3G–I). These data indicated that the serum‐derived EVs aggravated AF and atrial remodeling induced by Ang II by carrying MIAT.

### 3.4 miR‐485‐5p is a downstream molecule of MIAT and regulates CXCL10

**FIGURE 4:**
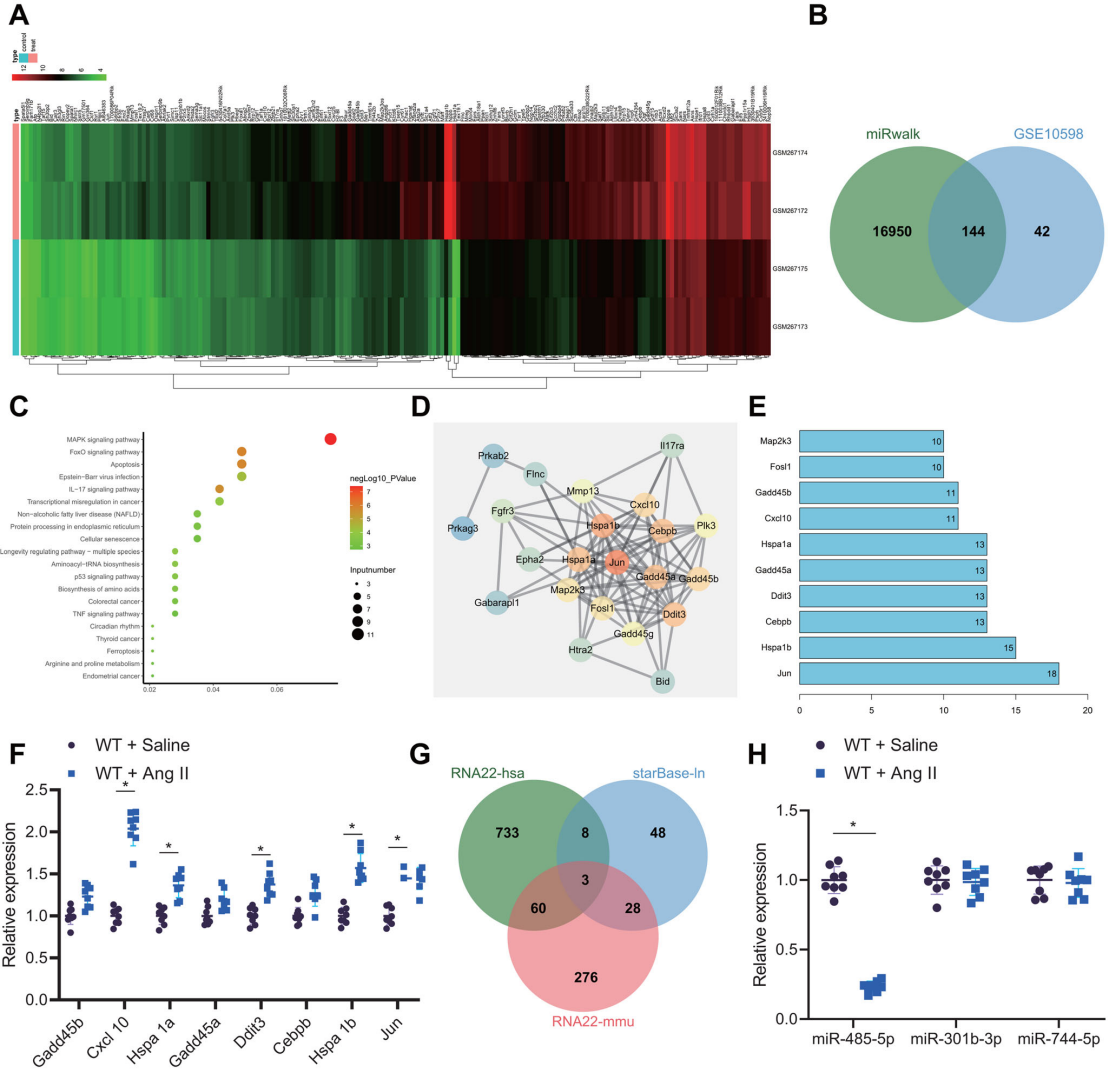
Bioinformatics analysis of target genes of miR‐485‐5p and differentially expressed genes in AF. (A) Plot of the significantly upregulated genes in AF samples in the AF‐related GSE10598 dataset. The abscissa represents the sample number, and the ordinate represents the differentially expressed genes; the left histogram shows gene expression cluster; each rectangle corresponds to a sample expression value; the histogram at the upper right refers to color gradation. (B) Intersection of the downstream genes of miRNA predicted using the miRWalk database and the upregulated genes identified in the GSE10598 dataset. The central part indicates the intersection of three sets of data. (C) KEGG enrichment analysis of the candidate intersected genes. The abscissa represents GeneRatio and the ordinate represents the name of KEGG items; the circle size indicates the number of genes enriched in the pathway; the color represents the enrichment *p*‐value, and the right histogram is the color scale. (D) Interaction analysis of the candidate target genes. Each circle in the figure represents a gene, and the line between circles indicates interaction between two genes; the deeper color of genes reflects higher the degree value and core degree. (E) Statistics of the degree value of core genes; the abscissa represents the degree value, and the ordinate represents the gene name. (F) Expression of eight candidate genes in atrial tissues of Ang II‐induced mice determined by RT‐qPCR. (G) Intersection of the upstream miRNAs of CXCL10 in human and mice predicted by the RNA22 database and the downstream miRNAs of MIAT predicted by the starBase database. The central part indicates the intersection of three sets of data. (H) Expression of three candidate genes in atrial tissues of Ang II‐induced mice determined by RT‐qPCR. *n* = 8 for mice upon each treatment. *p < .05. Data between two groups were analyzed by unpaired *t*‐test

To have further knowledge of the specific regulatory mechanism of MIAT delivered by serum‐derived EVs, the downstream miRNAs of MIAT in mice were presumed using the starBase database, which clarified 87 candidate miRNAs (Table S4). Next, target genes of the candidate miRNAs were presumed utilizing miRWalk database. At the same time, we performed differential analysis on the AF‐related dataset GSE10598 (contained two normal samples and two disease samples) retrieved from the GEO database utilizing R “limma” package with |logFC| > 1, adjusted *p*‐value <.05 as the threshold, and obtained 186 significantly upregulated genes in AF samples (Figure 4A). Next, these upregulated genes were intersected with the aforementioned target genes of miRNAs (Figure 4B), and then the yielded candidate genes were subjected to further KEGG enrichment analysis (Figure 4C) and gene interaction analysis (Figure 4D), with the degree values of core genes counted (Figure 4E). It was found that eight genes had the degree value greater than 10, and the quantitative analysis for these genes suggested the most significantly upregulated CXCL10 expression in atrial tissues of Ang II‐induced mice (Figure 4F). Notably, CXCL10 was signified to be abundantly expressed in AF.^15^ Therefore, CXCL10 was selected as the research subject. Then, predicted upstream miRNAs of CXCL10 in human and mice using RNA22 database were intersected with the predicted downstream miRNAs of MIAT. Finally, three miRNAs were obtained (Figure 4G). Quantitative analysis showed miR‐485‐5p downregulation in atrial tissues of Ang II‐induced mice (Figure 4H). Conclusively, MIAT might bind to miR‐485‐5p to manipulate CXCL10 in AF.

### 3.5 MIAT binds to miR‐485‐5p to upregulate CXCL10

**FIGURE 5:**
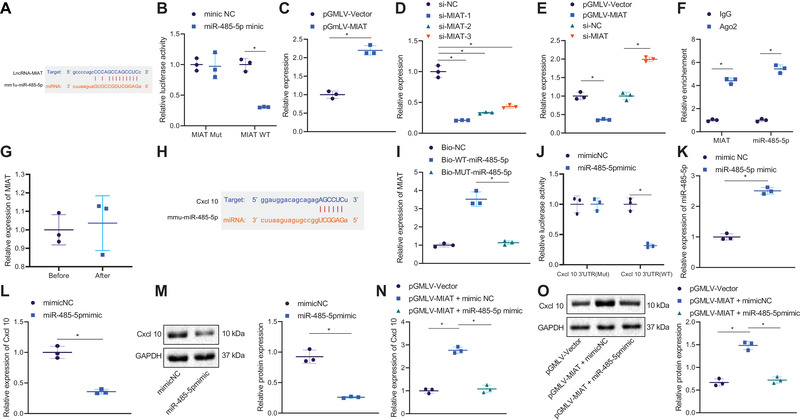
MIAT upregulates the expression of CXCL10 by binding to miR‐485‐5p. (A) Putative binding sites between MIAT and miR‐485‐5p predicted by the RNA22 database. (B) Binding of miR‐485‐5p to MIAT confirmed by dual‐luciferase reporter assay. (C) Transduction efficiency of pGMLV‐MIAT in HL‐1 cells determined by RT‐qPCR. (D) Transduction efficiency of si‐MIAT in HL‐1 cells determined by RT‐qPCR. (E) Expression of miR‐485‐5p in HL‐1 cells transfected with pGMLV‐MIAT or si‐MIAT determined by RT‐qPCR. (F) MIAT and miR‐485‐5p co‐immunoprecipitated by the Ago2 antibody determined by RIP. (G) Abundance of MIAT determined by RT‐qPCR in serum‐derived EVs before and after treatment with RNase. (H) Quantity of MIAT and miR‐485‐5p in the immune complexes determined by RNA pull‐down assay. (I) Putative miR‐485‐5p binding sites in the 3′UTR of CXCL10 mRNA predicted by the RNA22 database. (J) Binding of miR‐485‐5p to CXCL10 confirmed by dual‐luciferase reporter assay. (K) Transfection efficiency of miR‐485‐5p mimic in HL‐1 cells determined by RT‐qPCR. (L) mRNA expression of CXCL10 in miR‐485‐5p mimic‐transfected HL‐1 cells determined by RT‐qPCR. (M) Western blot analysis of CXCL10 protein in miR‐485‐5p mimic‐transfected HL‐1 cells. (N) mRNA expression of CXCL10 in HL‐1 cells transfected with pGMLV‐MIAT and/or miR‐485‐5p mimic determined by RT‐qPCR. (O) Western blot analysis of CXCL10 protein in HL‐1 cells transfected with pGMLV‐MIAT and/or miR‐485‐5p mimic. *p < .05. Data between two groups were analyzed by unpaired *t*‐test. Data among multiple groups were analyzed using one‐way ANOVA with Tukey's multiple comparisons test. Cell experiments were repeated three times independently

Next, we attempted to elucidate the molecular interactions among miR‐485‐5p, MIAT, and CXCL10 in AF. As manifested in Figure 5A, the RNA22 database predicted binding sites between MIAT and miR‐485‐5p. Furthermore, miR‐485‐5p mimic decreased the luciferase activity of MIAT‐WT (Figure 5B). HL‐1 cells were transduced with pGMLV‐MIAT and si‐MIAT, and the efficiency of both was confirmed by RT‐qPCR (Figure 5C,D). The si‐MIAT‐1 showed the superior silencing efficiency and was thus used for subsequent experiments. RT‐qPCR data showed upregulated miR‐485‐5p in HL‐1 cells after MIAT silencing but inhibited following MIAT overexpression (Figure 5E). In addition, RIP assay results showed that Ago2 antibody could coimmunoprecipitate both MIAT and miR‐485‐5p from cell lysate (Figure 5F). The serum‐derived EVs were treated with ribonuclease (RNase), and the abundance of MIAT was then determined by RT‐qPCR. The results displayed no significant difference in the abundance of MIAT before and after treatment (Figure 5G). Additionally, RNA pull‐down data revealed the binding of Bio‐WT‐miR‐485‐5p to MIAT rather than Bio‐MUT‐miR‐485‐5p (Figure 5H). Furthermore, the RNA22 database predicted miR‐485‐5p binding sites in 3′UTR of CXCL10 (Figure 5I), and meanwhile, the luciferase activity of CXCL10‐WT was found to be reduced following miR‐485‐5p mimic transfection (Figure 5J). Additionally, miR‐485‐5p expression (Figure 5K) was increased, whereas the CXCL10 mRNA and protein expression (Figure 5L,M) was diminished in miR‐485‐5p mimic‐transfected HL‐1 cells. Besides, an enhancement of CXCL10 expression was found in the presence of pGMLV‐MIAT, whereas this enhancement was nullified by miR‐485‐5p mimic (Figure 5N,O). In summary, MIAT could bind to miR‐485‐5p and thus upregulated CXCL10.

### 3.6 CXCL10 overexpression facilitates atrial fibrosis, inflammation, and oxidative stress

**FIGURE 6:**
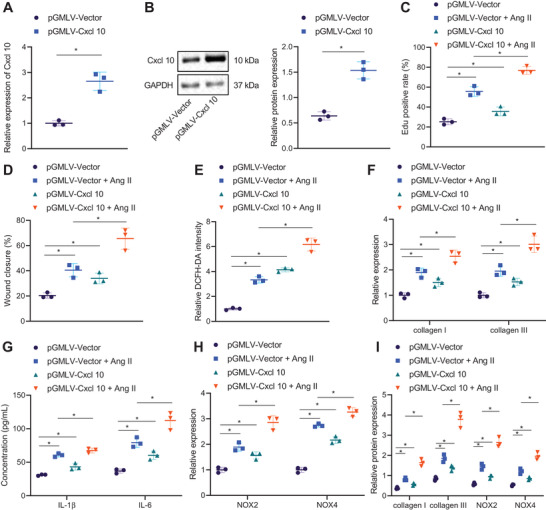
CXCL10 accelerates atrial fibrosis, inflammation, and oxidative stress. (A) Transduction efficiency of pGMLV‐CXCL10 in HL‐1 cells determined by RT‐qPCR. (B) Transduction efficiency of pGMLV‐CXCL10 in HL‐1 cells measured by Western blot analysis. HL‐1 cells were transfected with pGMLV‐Vector or pGMLV‐CXCL10, and Ang II‐induced HL‐1 cells were transfected with pGMLV‐Vector or pGMLV‐CXCL10. (C) Proliferation of HL‐1 cells measured by EdU assay. (D) Migration of HL‐1 cells measured by Scratch test. (E) Production of superoxide in HL‐1 cells measured by DCFH‐DA. (F) Expression of collagen I and collagen III determined by RT‐qPCR in HL‐1 cells. (G) Expression of IL‐1β and IL‐6 in the supernatant of HL‐1 cells detected by ELISA. (H) Expression of NOX2 and NOX4 determined by RT‐qPCR in HL‐1 cells. (I) Western blot analysis of collagen I, collagen III, NOX2, and NOX4 proteins in HL‐1 cells. *p < .05. Data between two groups were analyzed by unpaired *t*‐test. Data among multiple groups were analyzed using one‐way ANOVA with Tukey's multiple comparisons test. Cell experiments were repeated three times independently

We then focused on analyzing the role of CXCL10 in atrial fibrosis, inflammation, and oxidative stress. Upregulated CXCL10 was observed in HL‐1 cells transduced with pGMLV‐CXCL10 (Figure 6A,B). The proliferation (Figure 6C), migration (Figure 6D), and superoxide production (Figure 6E) of HL‐1 cells were elevated by treatment with pGMLV‐vector + Ang II or pGMLV‐CXCL10. Furthermore, the proliferation (Figure 6C), migration (Figure 6D), and superoxide production (Figure 6E) of HL‐1 cells induced by Ang II were accelerated following CXCL10 overexpression. In addition, pGMLV‐Vector + Ang II or pGMLV‐CXCL10 resulted in elevated collagen I, collagen III, IL‐1β, IL‐6, NOX2, and NOX4 expression in HL‐1 cells, while dual treatment with pGMLV‐CXCL10 and Ang II could further enhance the expression of these factors (Figure 6F–I). Conversely, si‐NC + Ang II treatment caused enhancement of proliferation (Figure S1C), migration (Figure S1D), and superoxide production (Figure S1E) of HL‐1 cells, which si‐CXCL10 contributed to opposite trends. In the presence of Ang II, CXCL10 diminished proliferation (Figure S1C), migration (Figure S1D), and superoxide production (Figure S1E) of HL‐1 cells. As reflected in Figure S1F–K, si‐NC + Ang II treatment augmented but si‐CXCL10 reduced collagen I, collagen III, IL‐1β, IL‐6, NOX2, and NOX4 expression in HL‐1 cells. Moreover, si‐CXCL10 reduced collagen I, collagen III, IL‐1β, IL‐6, NOX2, and NOX4 expression in Ang II‐induced HL‐1 cells. The above data demonstrated that upregulated CXCL10 could promote atrial fibrosis, inflammation, and oxidative stress.

### 3.7 Serum‐derived EVs stimulate atrial fibrosis, inflammation, and oxidative stress through MIAT/miR‐485‐5p/CXCL10 axis

**FIGURE 7:**
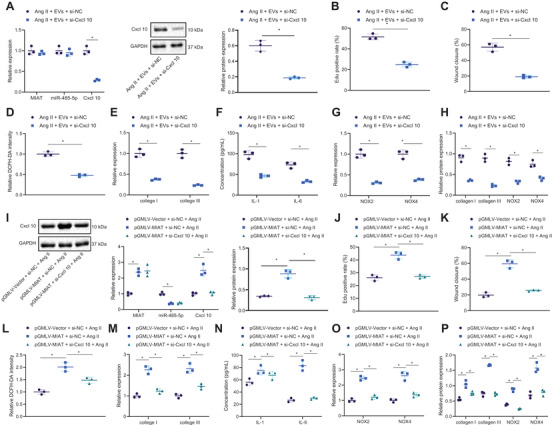
Serum‐derived EVs boost atrial fibrosis, inflammation, and oxidative stress via the MIAT/miR‐485‐5p/CXCL10 axis. (A–H) Ang II‐induced HL‐1 cells were treated with EVs + si‐NC or EVs + si‐CXCL10. (A) Expression of MIAT, miR‐485‐5p, and CXCL10 in HL‐1 cells determined by RT‐qPCR as well as CXCL10 protein expression in HL‐1 cells determined by Western blot analysis. (B) Proliferation of HL‐1 cells measured by EdU assay. (C) Migration of HL‐1 cells measured by Scratch test. (D) Production of superoxide in HL‐1 cells measured by DCFH‐DA. (E) Expression of collagen I and collagen III determined by RT‐qPCR in HL‐1 cells. (F) Expression of IL‐1β and IL‐6 in the supernatant of HL‐1 cells measured by ELISA. (G) Expression of NOX2 and NOX4 determined by RT‐qPCR in HL‐1 cells. (H) Western blot analysis of collagen I, collagen III, NOX2, and NOX4 in HL‐1 cells. (I–P) Ang II‐induced HL‐1 cells were treated with pGMLV‐Vector + si‐NC, pGMLV‐MIAT + si‐NC, or pGMLV‐MIAT + si‐CXCL10. (I) Expression of MIAT, miR‐485‐5p, and CXCL10 in HL‐1 cells determined by RT‐qPCR as well as CXCL10 protein expression in HL‐1 cells determined by Western blot analysis. (J) Proliferation of HL‐1 cells measured by EdU assay. (K) Migration of HL‐1 cells measured by Scratch test. (L) Production of superoxide in HL‐1 cells measured by DCFH‐DA. (M) Expression of collagen I and collagen III determined by RT‐qPCR in HL‐1 cells. (N) Expression of IL‐1β and IL‐6 in the supernatant of HL‐1 cells detected by ELISA. (O) Expression of NOX2 and NOX4 determined by RT‐qPCR in HL‐1 cells. (P) Western blot analysis of collagen I, collagen III, NOX2, and NOX4 proteins in HL‐1 cells. *p < .05. Data between two groups were analyzed by unpaired *t*‐test. Data among multiple groups were analyzed using one‐way ANOVA with Tukey's multiple comparisons test. Cell experiments were repeated three times independently

The following experiments focused at exploring whether serum‐derived EVs play a role through the MIAT/miR‐485‐5p/CXCL10 axis. First, si‐CXCL10 did not affect MIAT and miR‐485‐5p expression while reducing that of CXCL10 mRNA and protein in Ang II‐induced HL‐1 cells treated with EVs (Figure 7A). Additionally, CXCL10 silencing resulted in the reduced proliferation (Figure 7B), migration (Figure 7C), and superoxide production (Figure 7D) of Ang II‐induced HL‐1 cells treated with EVs. In addition, collagen I, collagen III, IL‐6, IL‐1β, NOX2, and NOX4 expression was diminished upon CXCL10 silencing in Ang II‐induced HL‐1 cells treated with EVs (Figure 7E–H). As documented in Figure 7I, MIAT and CXCL10 were upregulated but miR‐485‐5p was downregulated in Ang II‐induced HL‐1 cells treated with pGMLV‐MIAT + si‐NC, whereas MIAT and miR‐485‐5p expression showed no alterations while CXCL10 mRNA and protein expression was reduced in Ang II‐induced HL‐1 cells treated with pGMLV‐MIAT + si‐CXCL10 as compared to the Ang II‐induced HL‐1 cells treated with pGMLV‐MIAT + si‐NC. The increased proliferation (Figure 7J), migration (Figure 7K), and superoxide production (Figure 7L) of HL‐1 cells following pGMLV‐MIAT treatment could be abolished by CXCL10 knockdown in the Ang II‐induced HL‐1 cells. Furthermore, upregulated collagen I, collagen III, IL‐6, IL‐1β, NOX2, and NOX4 caused by pGMLV‐MIAT was also reversed by CXCL10 knockdown in the Ang II‐induced HL‐1 cells (Figure 7M–P). The above data indicated that serum‐derived EVs promoted atrial fibrosis, inflammation, and oxidative stress through MIAT/miR‐485‐5p/CXCL10 axis.

### 3.8 Serum‐derived EVs accelerate Ang II‐caused AF and atrial remodeling via MIAT/miR‐485‐5p/CXCL10 axis in vivo

**FIGURE 8:**
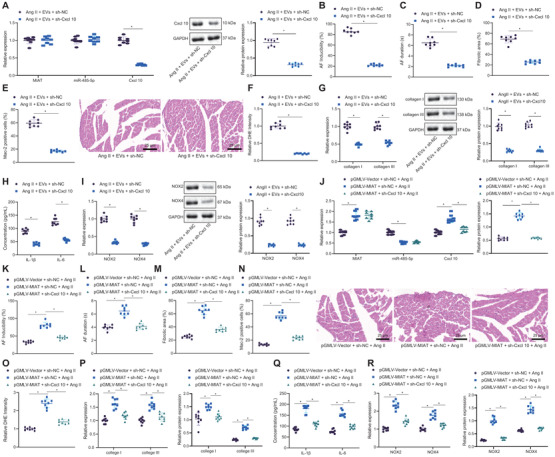
Serum‐derived EVs promote AF and atrial remodeling via the MIAT/miR‐485‐5p/CXCL10 axis in vivo. (A–I) Ang II‐induced mice were treated with serum‐derived EVs + si‐NC or serum‐derived EVs + si‐CXCL10. (A) Expression of MIAT, miR‐485‐5p, and CXCL10 determined by RT‐qPCR in atrial tissues of Ang II‐induced mice as well as CXCL10 protein expression determined by Western blot analysis in atrial tissues of Ang II‐induced mice. (B) AF induction rate of Ang II‐induced mice. (C) AF duration of Ang II‐induced mice. (D) Masson's trichrome staining of fibrosis in atrial tissues of Ang II‐induced mice. (E) HE staining of pathological situations in atrial tissues of Ang II‐induced mice (scale bar = 25 μm), and immunohistochemistry for Mac‐2 positive cells in atrial tissues of Ang II‐induced mice. (F) DHE staining of superoxide production in atrial tissues of Ang II‐induced mice. (G) Expression of collagen I and collagen III determined by RT‐qPCR in atrial tissues of Ang II‐induced mice. (H) Expression of IL‐1β and IL‐6 in atrial tissues of Ang II‐induced mice measured by ELISA. (I) Expression of NOX2 and NOX4 determined by RT‐qPCR in atrial tissues of Ang II‐induced mice. (J–R), Ang II‐induced mice treated with pGMLV‐Vector + si‐NC, pGMLV‐MIAT + si‐NC, or pGMLV‐MIAT + si‐CXCL10. (J) Expression of MIAT, miR‐485‐5p, and CXCL10 determined by RT‐qPCR in atrial tissues of Ang II‐induced mice as well as CXCL10 protein expression determined by Western blot analysis in atrial tissues of Ang II‐induced mice. (K) AF induction rate of Ang II‐induced mice. (L) AF duration of Ang II‐induced mice. (M) Masson's trichrome staining of fibrosis in atrial tissues of Ang II‐induced mice. (N) HE staining of pathological situations in atrial tissues of Ang II‐induced mice (scale bar = 25 μm), and immunohistochemistry for Mac‐2‐positive cells in atrial tissues of Ang II‐induced mice. (O) DHE staining of superoxide production in atrial tissues of Ang II‐induced mice. (P) Expression of collagen I and collagen III determined by RT‐qPCR in atrial tissues of Ang II‐induced mice. (Q) Expression of IL‐1β and IL‐6 in atrial tissues of Ang II‐induced mice measured by ELISA. (R) Expression of NOX2 and NOX4 determined by RT‐qPCR and Western blot analysis in atrial tissues of Ang II‐induced mice. *n* = 8 for mice upon each treatment. *p < .05. Data among multiple groups were analyzed using one‐way ANOVA with Tukey's multiple comparisons test

Finally, we aimed to characterize the effect of serum‐derived EVs on the AF and atrial remodeling by the MIAT/miR‐485‐5p/CXCL10 axis in vivo. No change in MIAT and miR‐485‐5p expression was found while CXCL10 expression was decreased by treatment with si‐CXCL10 in atrial tissues of Ang II‐induced mice treated with EVs (Figure 8A). SBP of Ang II‐induced mice was decreased following dual treatment with EVs and si‐CXCL10. Besides, SBP of Ang II‐induced mice was increased upon CXCL10 silencing in the presence of EVs (Figure S1B). Moreover, AF induction rate and AF duration were noted to be decreased by CXCL10 knockdown in Ang II‐induced mice treated with EVs (Figure 8B,C). In addition, CXCL10 knockdown suppressed the atrial fibrosis (Figure 8D), inflammatory cell infiltration and Mac‐2‐positive macrophages (Figure 8E), and superoxide production (Figure 8F) in Ang II‐induced mice treated with EVs. Meanwhile, CXCL10 knockdown downregulated collagen I, collagen III, IL‐6, IL‐1β, NOX2, and NOX4 in atrial tissues of Ang II‐induced mice treated with both EVs (Figure 8G–I). Treatment with pGMLV‐MIAT elevated MIAT and CXCL10 expression but reduced miR‐485‐5p expression in atrial tissues of Ang II‐induced mice, while treatment with si‐CXCL10 reduced the expression of CXCL10 without affecting MIAT and miR‐485‐5p expression in atrial tissues of Ang II‐induced mice treated with pGMLV‐MIAT (Figure 8J). Additionally, SBP of Ang II‐induced mice was increased upon MIAT overexpression while further CXCL10 silencing reduced the SBP (Figure S1B). CXCL10 knockdown reversed the promoting effects of pGMLV‐MIAT on AF induction rate and AF duration in Ang II‐induced mice (Figure 8K,L). Additionally, CXCL10 knockdown counteracted the promoting effects of pGMLV‐MIAT on atrial fibrosis, inflammatory cell infiltration and Mac‐2‐positive macrophages, superoxide production, and expression of collagen I, collagen III, IL‐6, IL‐1β, NOX2, and NOX4 in atrial tissues in Ang II‐induced mice (Figure 8M–R). Cumulatively, the serum‐derived EVs exerted promoting properties on the AF and atrial remodeling via the MIAT/miR‐485‐5p/CXCL10 axis in vivo.

## 4 DISCUSSION

AF is a prevalent arrhythmia in the intensive care unit, which can be triggered by atrial remodeling and arrhythmogenic triggers.^20^ The findings collected from the present study supported the promoting effect of MIAT shuttled by serum‐derived EVs on the atrial remodeling and the resultant AF by disrupting miR‐485‐5p‐mediated CXCL10 inhibition.

The present study revealed abundant MIAT expression in serum‐derived EVs of patients with AF and that serum‐derived EVs could carry MIAT to promote atrial fibrosis, inflammation, and oxidative stress. As small membranous vesicles released by various cell types, EVs assume a central role in cell‐to‐cell communication through delivering ncRNAs like miRNAs, lncRNAs, and circular RNAs.^21^ EV‐mediated ncRNA transfer participates in human diseases, especially in cancer where they play important roles in manipulating the pivotal genes that triggers tumorigenesis, angiogenesis, metastasis, immunity, and drug resistance.^22^ Serum‐derived EVs have been extensively recognized to have the potential to serve as diagnostic and prognostic biomarkers for a variety of human diseases, such as sclerosing cholangitis, cholangiocarcinoma, and colon cancer.^23^, ^24^ Partially in accordance with this finding, MIAT expression has been detected to be upregulated in the exosomes derived from serum samples of gastric cancer patients, and the serum exosomal MIAT can accelerate the progression of this cancer.^8^ Additionally, the expression of MIAT shows a substantial increase in atrial tissues of AF rats while MIAT downregulation contributes to the alleviation of AF and AF‐induced myocardial fibrosis, which is evidenced by the reduced expression of fibrotic markers collagen I and collagen III.^10^ The existing evidence has marked that the progression of atrial fibrosis is inseparable from myofibroblast differentiation. Fibroblasts can differentiate into myofibroblasts, accompanied by upregulated expression of collagen I and transforming growth factor‐β1 (TGF‐β1) and alpha‐smooth muscle actin synthesis, whose assembly has become crucial directions in the mechanistic study of atrial fibrosis.^25^ It was documented in a prior work that MIAT was able to orchestrate TGF‐β1,^26^ which reported MIAT as a potent mediator in myofibroblast differentiation. However, it needs further exploration about whether MIAT impacts myofibroblast differentiation to manipulate atrial fibrosis. A recent study has indicated that nicotine‐mediated MIAT induction is responsible for excessive cardiac collagen deposition.^27^ Another study has also demonstrated the proinflammatory and pro‐oxidative effect of MIAT in lipopolysaccharide (LPS)‐induced septic cardiomyopathy.^28^

Studies have confirmed that MIAT can act as miRNA sponges and thus impair the regulatory effects of miRNAs on the target mRNAs.^29^, ^30^ The promoting effect of MIAT on the inflammation response and oxidative stress has been shown to be achieved by direct binding to miR‐330‐5p and activating the TRAF6/NF‐κB signaling.^28^ In this study, MIAT was identified to bind to miR‐485‐5p and upregulated CXCL10, a target of miR‐485‐5p. miR‐485‐5p possesses the capacity to block cardiac hypertrophy induced by phenylephrine and confers protection of cardiac structure and function.^13^ In addition, miR‐485‐5p augments cardiomyocyte proliferation and represses cell apoptosis in H_2_O_2_‐caused myocardial oxidative injury.^31^ Also, miR‐485‐5p has been suggested to downregulate oxidative markers and hence protects against ischemia‐reperfusion injury.^32^ However, the probable role of miR‐485‐5p in the atrial myocytes following AF is not fully elucidated. Elevated CXCL10 expression can be observed in AF patients,^15^ which is in accordance to our results. Additionally, CXCL10 has been recognized to be a marker of oxidative stress and inflammation,^33^ and its inhibition in astrocytes and microglia/macrophages aids in the attenuation of oxidative stress‐induced inflammation following ischemia‐reperfusion injury.^34^ Meanwhile, CXCL10 can significantly upregulate the expression of collagen I in human dermal fibroblasts.^35^ These findings are in agreement with ours that CXCL10 overexpression could facilitate atrial fibrosis, inflammation, and oxidative stress. We can thus reach a conclusion that serum‐derived EVs could drive atrial fibrosis, inflammation, and oxidative stress through regulation of the MIAT/miR‐485‐5p/CXCL10 signaling axis.

## 5 CONCLUSION

**FIGURE 9:**
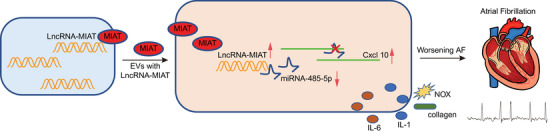
Schematic diagram of the mechanism by which serum‐derived EVs affect AF via the MIAT/miR‐485‐5p/CXCL10 axis. EVs isolated from serum samples of AF patients carried MIAT to bind to miR‐485‐5p and caused the upregulation of the miR‐485‐5p target CXCL10, thus aggravating the AF

Overall, our study indicates that serum‐derived EVs can potentially carry MIAT, which binds to miR‐485‐5p and weakens its suppressive role on the target CXCL10, thus promoting the atrial remodeling and the consequent AF (Figure 9). Therefore, EV‐mediated transfer of MIAT may act as a specific and sensitive biomarker for diagnosing and monitoring the progression of AF. However, the established association of MIAT expression with EVs lacks evidence to support, and so further investigations should be performed to ensure therapeutic efficacy in clinics. In addition, EV purification is a highly controversial field, and the method for the generation of a contamination‐free EV suspension is still missing. The ultracentrifugation method used for EV isolation in this study might lead to contamination of the isolate with lipoproteins and extracellular nucleic acids. Co‐isolated non‐EV material from serum samples may also cause influence on the delivered MIAT abundance by EVs. Meanwhile, additional studies are warranted to determine whether RNA and other RNA species are actually associated to EVs or only co‐isolated.

## CONFLICT OF INTEREST

The authors declare that there is no conflict of interest.

## Supporting information


**Supporting Figure** S1 Silencing of CXCL10 attenuates SBP, atrial fibrosis, inflammation, and oxidative stress. (A) SBP of Ang II‐induced mice treated with EVs or pGMLV‐MIAT. (B) SBP of Ang II‐induced mice following treatment with EVs + si‐NC, si‐CXCL10 + EVs, pGMLV‐Vector, pGMLV‐MIAT, or pGMLV‐MIAT + si‐CXCL10. (C) Transduction efficiency of si‐CXCL10 in HL‐1 cells determined by RT‐qPCR. (D) Transduction efficiency of si‐CXCL10 in HL‐1 cells determined by Western blot analysis. HL‐1 cells were transduced with si‐NC or si‐CXCL10, and Ang II‐induced HL‐1 cells were transduced with si‐NC or si‐CXCL10. (E) Proliferation of HL‐1 cells measured by EdU assay. (F) Migration of HL‐1 cells measured by Scratch test. (G) Production of superoxide in HL‐1 cells measured by DCFH‐DA. (H) Expression of collagen I and collagen III determined by RT‐qPCR in HL‐1 cells. (I) Expression of IL‐1β and IL‐6 in the supernatant of HL‐1 cells detected by ELISA. (J) Expression of NOX2 and NOX4 determined by RT‐qPCR in HL‐1 cells. (K) Western blot analysis of collagen I, collagen III, NOX2, and NOX4 proteins in HL‐1 cells. *p < .05. Data between two groups were analyzed by unpaired *t*‐test. Data among multiple groups were analyzed using one‐way ANOVA with Tukey's multiple comparisons test.

SUPPORTING INFORMATION

SUPPORTING INFORMATION

SUPPORTING INFORMATION

SUPPORTING INFORMATION

## Data Availability

The datasets generated and/or analyzed during the current study are available from the corresponding author upon reasonable request.
