# Supplementary material for: Serum extracellular vesicles containing MIAT induces atrial fibrosis, inflammation and oxidative stress to promote atrial remodeling and atrial fibrillation via blockade of miR‐485‐5p‐mediated CXCL10 inhibition
Source: Clin Transl Med. 2021 Aug 3;11(8):e482. doi: 10.1002/ctm2.482 (PMC8329545; doi:10.1002/ctm2.482)
Supplement: Supplementary file 2 — SUPPORTING INFORMATION [file CTM2-11-e482-s004.docx]

**Supplementary Table 1** Clinical characteristics of atrial fibrillation patients and normal volunteers

|  | Atrial fibrillation | Normal | *p* value |
| --- | --- | --- | --- |
| Number of patients | 20 | 20 |  |
| Mean age (SD, years) | 58.90 ± 6.32 | 59.50 ± 5.77 | > 0.05 |
| Gender (male/female) | 14/6 | 15/5 | > 0.05 |
| Smoking history (yes) | 14 | 11 | > 0.05 |
| Left atrial diameter | 29.47 ± 2.05 | 45.85 ± 7.12 | > 0.05 |
| Left ventricular ejection fraction | 53.73 ± 2.82 | 64.29 ± 5.71 | > 0.05 |
| Systolic blood pressure (mmHg) | 121.80 ± 12.27 | 146.55 ± 14.08 | > 0.05 |
| Diastolic blood pressure (mmHg) | 74.15 ± 5.80 | 97.70 ± 8.13 | > 0.05 |
