# Supplementary material for: Serum extracellular vesicles containing MIAT induces atrial fibrosis, inflammation and oxidative stress to promote atrial remodeling and atrial fibrillation via blockade of miR‐485‐5p‐mediated CXCL10 inhibition
Source: Clin Transl Med. 2021 Aug 3;11(8):e482. doi: 10.1002/ctm2.482 (PMC8329545; doi:10.1002/ctm2.482)
Supplement: Supplementary file 3 — SUPPORTING INFORMATION [file CTM2-11-e482-s003.docx]

**Supplementary Table 2** Primer sequences for reverse transcription quantitative polymerase chain reaction

| Target | Primer sequence |
| --- | --- |
| Mouse miR-485-5p | Forward 5’-AGAGGCTGGCCGTGATG-3’ |
|  | Reverse 5’-GTGCTAGGGTCCGAGGT-3’ |
| U6 | Forward 5’-GCTTCGGCAGCACATATACTAAAAT-3’ |
|  | Reverse 5’-CGCTTCACGAATTTGCGTGTCAT-3’ |
| Mouse miR-301b-3p | Forward 5’-GCGCAGTGCAATGGTATTGT-3’ |
|  | Reverse 5’-AGTGCAGGGTCCGAGGTATT-3’ |
| Mouse miR-744-5p | Forward 5’-ATACTCGAGATCCTAGTTTGATACTCCCAGTCTT-3’ |
|  | Reverse 5’-TGTTCTAGACATATTTACTTTTATATTTCCATAC-3’ |
| cel-miR-39 | Forward 5’-GGGGAGCTGATTTCGTCTTG-3’ |
|  | Reverse 5’-CTCAACTGGTGTCGTGGAGT-3’ |
| Mouse MIAT | Forward 5’-AGGTGCTGGAATTCCGTCTG-3’ |
|  | Reverse 5’- GCAGGCTAGTCGTGACCTTT -3’ |
| Human MIAT | Forward 5’- AAGCAGGAAGCTCACACCTC -3’ |
|  | Reverse 5’- CCACAGACCCCTGACCAATC -3’ |
| Human GAPDH | Forward 5’- AATGGGCAGCCGTTAGGAAA -3’ |
|  | Reverse 5’- AGTTAAAAGCAGCCCTGGTGA -3’ |
| Mouse collagen I | Forward 5’-TGACTGGAAGAGCGGAGAGTACT-3’ |
|  | Reverse 5’-TTCGGGCTGATGTACCAGTTC-3’ |
| Mouse collagen III | Forward 5’-AAATTCTGCCACCCCGAACT-3’ |
|  | Reverse 5’-CCAGTGCTTACGTGGGACAGT-3’′ |
| Mouse NOX2 | Forward 5’-GCTACGCCTTCAACACCAAG-3’ |
|  | Reverse 5’-AGTTCGTCCCCTTCTCCTGT-3’ |
| Mouse NOX4 | Forward 5’-GCACGCTGTTGATTTTTATGG-3’ |
|  | Reverse 5’-GCGAGGCAGGAGAGTCAGTA-3’ |
| Mouse CXCL10 | Forward 5’-CCAAGTGCTGCCGTCATTTTC-3’ |
|  | Reverse 5’-TCCCTATGGCCCTCATTCTCA-3’ |
| Mouse Gadd45b | Forward 5’-GAGGCGGCCAAACTGATGAAT-3’ |
|  | Reverse 5’-CGCAGCAGAACGACTGGAT-3’ |
| Mouse Hspa1a | Forward 5’-TGGTGCAGTCCGACATGAAG-3’ |
|  | Reverse 5’-GCTGAGAGTCGTTGAAGTAGGC-3’ |
| Mouse Gadd45a | Forward 5’-AGACCGAAAGGATGGACACG-3’ |
|  | Reverse 5’-GTACACGCCGACCGTAATG-3’ |
| Mouse Ddit3 | Forward 5’-AAGCCTGGTATGAGGATCTGC-3’ |
|  | Reverse 5’-TTCCTGGGGATGAGATATAGGTG-3’ |
| Mouse Cebpb | Forward 5’- TTATAAACCTCCCGCTCGGC -3’ |
|  | Reverse 5’- AGTCGGGCTCGTAGTAGAAGT -3’ |
| Mouse Hspa1b | Forward 5’-GAGATCGACTCTCTGTTCGAGG-3’ |
|  | Reverse 5’-GCCCGTTGAAGAAGTCCTG-3’ |
| Mouse Jun | Forward 5’-ACTCGGACCTTCTCACGTC-3’ |
|  | Reverse 5’-GGTCGGTGTAGTGGTGATGT-3’ |
| Mouse GAPDH | Forward 5’-GTGTTTCCTCGTCCCGTAGA-3’ |
|  | Reverse 5’-AATCTCCACTTTGCCACTGC-3’ |
