# Supplementary material for: Serum extracellular vesicles containing MIAT induces atrial fibrosis, inflammation and oxidative stress to promote atrial remodeling and atrial fibrillation via blockade of miR‐485‐5p‐mediated CXCL10 inhibition
Source: Clin Transl Med. 2021 Aug 3;11(8):e482. doi: 10.1002/ctm2.482 (PMC8329545; doi:10.1002/ctm2.482)
Supplement: Supplementary file 4 — SUPPORTING INFORMATION [file CTM2-11-e482-s001.docx]

**Supplementary Table 3** Clinical characteristics of patients with atrial fibrillation subtypes and MIAT expression in the EVs from serum samples of these patients

|  | Paroxysmal atrial fibrillation | Persistent atrial fibrillation | Permanent atrial fibrillation | *p* value |
| --- | --- | --- | --- | --- |
| Number of patients | 6 | 7 | 7 |  |
| Mean age (SD, years) | 53.50 ± 3.94 | 58.71 ± 3.59 | 65.43 ± 1.40 | < 0.05 |
| Gender (male/female) | 4/2 | 5/2 | 6/1 | > 0.05 |
| Smoking history (yes) | 3 | 5 | 3 | > 0.05 |
| Left atrial diameter | 38.41 ± 1.68 | 43.75 ± 2.46 | 54.34 ± 2.70 | < 0.05 |
| MIAT expression | 2.908 ± 0.103 | 2.986 ± 0.216 | 3.448 ± 0.111 | < 0.05 |
| Left ventricular ejection fraction | 69.89 ± 3.19 | 64.47 ± 0.35 | 59.30 ± 5.86 | < 0.05 |
| Systolic blood pressure (mmHg) | 133.67 ± 7.66 | 144.71 ± 10.27 | 159.43 ± 10.49 | < 0.05 |
| Diastolic blood pressure (mmHg) | 91.50 ± 6.75 | 96.29 ± 8.04 | 104.43 ± 3.31 | < 0.05 |
